# Testing the psychometric properties of a Chinese version of Dyadic Relationship Scale for families of people with hypertension in China

**DOI:** 10.1186/s40359-022-00747-2

**Published:** 2022-02-21

**Authors:** Dejian Zeng, Chen Yang, Wai Tong Chien

**Affiliations:** 1 Department of Nursing, School of Medical Technology and Nursing, Shenzhen Polytechnic, Shenzhen, China; 2 The Nethersole School of Nursing, The Chinese University of Hong Kong, Sha Tin, Hong Kong SAR China

## Abstract

**Background:**

Interventions for people with chronic illness have increasingly got involvement and partnership with family members in China and worldwide. The patient-family interactions in chronic illness care can greatly influence not only family dyadic relationship or collaboration in caregiving but also both patient’s and caregiver’s health and well-being. To date, very few instruments have been developed to measure the family dyadic relationship; and none has been found in Chinese language. This study aimed to translate the original English Dyadic Relationship Scales (DRS), including DRS-patient and DRS-caregiver, into simplified Chinese language (DRS-C) and examine their psychometric properties in Chinese people with hypertension in a rural community.

**Methods:**

The Brislin’s model of translation was adopted for translation. Face and content validity and semantic equivalence of the translated Chinese version of the two DRS-patient and DRS-caregiver scales were examined. A sample of 132 adults with hypertension and their family caregivers were recruited to test the psychometric properties of the DRS-C scales.

**Results:**

The DRS-C scales indicated very satisfactory face validity with 10 family dyads in hypertension care, content validity rated by five experts (Item CVI = 0.8–1.0; Scale CVI = 0.98) and semantic equivalence rated by 22 panel members (i.e., all items were rated as 3 = relevant or 4 = very relevant by > 18 members). A well-fitting model of DRS-C-patient was identified with χ^2^/df = 1.47, *p* = 0.04, RMSEA = 0.06, GFI = 0.941, CFI = 0.961, TLI = 0.947, and SRMR = 0.019. A well-fitting model of DRS-C-caregiver was identified with χ^2^/df = 1.340, *p* = 0.092, RMSEA = 0.039, GFI = 0.940, CFI = 0.975, TLI = 0.965, and SRMR = 0.014. The Chinese DRS-patient and DRS-caregiver had satisfactory internal consistency with Cronbach’s α coefficients of 0.82 and 0.83, respectively, and test–retest reliabilities with intra-correlation coefficients of 0.97 and 0.96, respectively. The convergent validities of the Chinese versions of the DRS-patient and DRS-caregiver were very satisfactory with the self-efficacy subscale of Hypertension Self-Care Profile, and Zarit Burden Interview, respectively (Pearson’s r = − 0.70 and 0.79; both *p* < 0.001). Significant differences on mean scores of the Chinese versions of the DRS-patient (t = − 8.10, *p* < 0.001) and the DRS-caregiver (t = − 9.15, *p* < 0.001), between the groups of adults with hypertension and normal blood pressure counterparts.

**Conclusion:**

Both Chinese versions of the DRS-patient and DRS-caregiver have sound psychometric properties and similar factor structure to the original English version. The Chinese versions can be valid measures of family dyadic relationship among Chinese adults with hypertension.

**Supplementary Information:**

The online version contains supplementary material available at 10.1186/s40359-022-00747-2.

## Introduction

According to the recommendations of the International Society of Hypertension and Chinese guidelines for the management of hypertension, hypertension is diagnosed when the office/clinic systolic blood pressure (SBP) is 140 mmHg or above and/or the diastolic blood pressure (DBP) is 90 mmHg or above [1, 2]. Hypertension is a major risk factor for cardio-cerebrovascular disease and its morbidity and mortality; and the increased risk of cardiovascular disease among adults with BP above 140/90 mmHg is well established [3].

Pharmacological and lifestyle modifications are the two main treatments for hypertension [1–3]. Hypertension management is an everyday, long-term process that needs continuous help and support from health care providers and family. Interventions involving family members are widely adopted in chronic illness care (e.g., stroke and diabetes) and hypertension management, especially in the community care settings [3, 4]. Patients with more support from family caregivers may show higher improved health-related knowledge, self-efficacy, better adherence behaviors, quality of life, and other important health outcomes [3, 4]. For family caregivers, they may have reduced caregiving burden and improved satisfaction with family care when they have shown good relationships with patients and other family members [4, 5]. On the other hand, if having a poor patient-family relationship, family caregiving can induce high burden of care and significant negative impacts on both patients’ and their caregivers’ physical and psychological health [6]. For example, the family dyads (patients and family caregivers) may feel very stressful in caregiving process and negative interpersonal interactions and relationships such as criticisms and nagging in communication, blaming and guilty feelings, and conflicts among family members [6, 7]. There would also be challenges in decision making on illness management and even over-controlling and protective behaviors to patients [8–10].

Indeed, most studies in chronic illness care have focused on either patients’ or caregivers’ interventions and outcomes. As a result, the interactive and reciprocal influence between the family dyads may often be neglected or underestimated. To measure the quality of this family dyadic relationship in hypertensive care, a psychometrically sound instrument should be available for an accurate and valid assessment [11, 12].

In China and worldwide, family care for people with hypertension has been an increasing important health care research topic. A systematic review of 94 randomized controlled trials evaluated their effectiveness of interventions for hypertension care in different Chinese communities, of which 13 studies used psychosocial interventions to improve family support [13]. Particularly, Chinese family culture and values highly emphasize the responsibilities and obligations of family members in caring for sick family members, considering to be the moral obligation and norms of Chinese societies [14]. Family caregivers often devote most of their time and efforts to ensure that the ill relatives can obtain adequate and appropriate medical treatment and assistance in daily living activities.

To date, very few instruments have been developed to measure patient and family or caregiver relationship, and none available in Chinese language. Dyadic Relationship Scale (DRS), including two independent scales (DRS-patient and DRS-caregiver versions), was the sole instrument measuring dyadic relationship in family care for people with chronic illnesses [15]. The DRS-patient and DRS-caregiver measure respective patient and caregiver viewpoints about how family caregiving to their patients impacts the interpersonal interactions between family members. Both DRS scales have demonstrated satisfactory internal consistency and good construct and concurrent validities among families of people with chronic illnesses in the United States [15]. For example, the Cronbach’s alpha coefficients of the DRS-patient and DRS-caregiver were 0.86 and 0.89, respectively. In addition, a best-fit model of two factors (i.e., positive interaction and dyadic strain) was identified with confirmatory factor analysis, with a root mean square error of approximation (RMSEA) of 0.07 and a comparative fit index (CFI) of 0.96. Concurrent validity was supported in the structural equation modelling and the partial standardized regression coefficient between the Centre for Epidemiologic Studies–Depression scale (CES-D) and the ‘dyadic strain’ subscale scores of the DRS-patient and DRS-caregiver were 0.53 and 0.47, respectively, both *p* < 0.05 [15].

The DRS scales are valid and useful to measure the quality of the perceived dyadic relationship between patients with chronic illness and their family caregivers [12].

For measuring such family dyad relationship in Chinese communities, this validation study aimed to translate the English DRS scales into simplified Chinese language followed by face and content validity and semantic equivalence testing, and to examine psychometric properties of the two translated Chinese version in Chinese families of people with hypertension.

## Methods

This study consisted of two phases: translation of the original DRS into the simplified Chinese language; and a descriptive survey study was conducted to examine psychometric properties of the translated Chinese versions.

### Phase one: translation process of the DRS, testing of face and content validity and semantic equivalence

The Brislin’s model of translation (and back-translation) was adopted to translate the original English DRS scales into simplified Chinese [16]. The translation process included the following steps: (1) *Forward translation*. Two bilingual Doctor of Philosophy (Ph.D.) students who were native Chinese speakers and registered nurses, translated the instrument from the source (English) language into the target (simplified Chinese) language. By discussion and consensus between the two translators, amendments were made before the revised simplified Chinese version was ready for back-translation process; (2) *Back-translation*. A bilingual nursing teacher with a Ph.D. degree blindly (without access to the original language version) back translated the revised Chinese version into English. Two translators then compared and discussed about any inconsistencies in meanings of the translated items. If disagreements on translated items and terminologies not resolved between two translators, the third and fourth translator would translate and back-translate the disagreed items, respectively, to seek agreements and finalized the items among the four translators.

The DRS-C scales were then tested with 10 family dyads of people with hypertension conveniently recruited from one village clinic to examine their face validity. Their comments on the items’ relevance to the study topic and context were collected through face-to-face interviews.

A panel of five experts, consisting of three academic experts on chronic illness management and two clinical nurses with more than 2 years of clinical experience in chronic illness care in the Chinese communities, was invited to rate the items of the DRS-C scales for their relevance to the construct of family dyadic relationship. A four-point Likert scale (1 = not relevant to 4 = highly relevant) was adopted to the rating of the level of relevance [17]. Content validity index (CVI) was used to evaluate the content validity of DRS-C at the item and scale levels [18]. The values of I-CVI ≥ 0.78 and S-CVI ≥ 0.90 were considered acceptable values for content validity of the DRS-C scales [17].

Furthermore, semantic equivalence of the DRS-C scales with the original scales was established by using the cross-language testing method [19]. Before using an instrument in a new language, cross-cultural validation should be performed. Family values in Chinese and Western cultures (e.g., Americans) are different [20]. These cultural specificities can influence family members’ or carers’ responses to the needs of their patients, self-efficacy of an ill relative and relationship of the family dyad in daily care [20]. The positive results of semantic equivalence computed by a group of bilingual participants can support the cultural relevance of the DRS-C scales to the Chinese population or culture. According to this method, the original and translated versions of the scales were administered to a group of bilingual participants. Semantic equivalence could be achieved when a high correlation by items was observed between the item scores of the two versions. However, the family carers and people with hypertension in the rural communities of Mainland China were unable to read and understand the English version of the DRS.

In this study, a panel of 22 members (six bilingual nursing researchers, 14 Ph.D. students in nursing, and two clinical nurses with master’s degree in nursing) were invited to rate the semantic equivalence of the translated DRS-C scales. Individual items in the DRS scales were rated on a four-point Likert scale in an ascending level of equivalence (“1 = Not Appropriate” to “4 = Most Appropriate”). An item was considered not equivalent if more than 20% of the panel members (i.e., at least four of the 22 panel members) rated the item as < 3 point according to the 4-point Likert scale used [17]. The non-equivalent item(s) would be revised by re-run of the translation and back-translation process described above.

### Phase two: psychometric testing of the two DRS-C scales

A cross-sectional descriptive study with correlational design, together with a re-test in 40 randomly selected participants over a two-week interval, was carried out to test the reliability and validity of the two DRS-C scales. A sample of 132 people with hypertension and their family caregivers living in a village in China were recruited in the current study.

### Participants and study setting

People with hypertension receiving home visit and care at a village clinic at Liuyang City Hunan Province China were the potential eligible participants of this study. The village clinic is a public clinic, providing primary health care to all people in the village. The researcher obtained permission from the clinic manager to review the patients’ medical records in the village clinic under study and created a list of potential participants after screening.

The patients on the list were approached by the researcher during home visits to confirm the eligibility of participants and collect data. Included patients were: diagnosed as an essential hypertension (SBP ≥ 140 mmHg and/or DBP ≥ 90 mmHg), with or without adequate BP control [2]; ≥ 18 years old; and living together with one or more family members. The patients were excluded if they were: with terminal illness; with mental illness (e.g., dementia and schizophrenia); having one or more comorbidities of severe cardiovascular cerebral and respiratory diseases (e.g., stroke, COPD and myocardial infarction); or having needs for assistance with daily activities, like toileting, feeding, dressing, grooming, physical ambulation or bathing.

The researcher guided the patients to nominate one main caregiver from each family to be the participants in this validation. The family carer should be the one who had provided more assistance with the patient’s daily health care and stayed a longer time with the patient in daily life among family members. Additional inclusion criteria of family carers were aged 18 years or above; and with kinship, marital or co-residence relationship with the patient. The carers were excluded if they were diagnosed with mental illness (e.g., schizophrenia and depression) or learning disorder; or taking care of two or more patients in the family at recruitment. Illiterates could be included since the questions of the questionnaire would be read out by the researcher for completion unless they were unable to understand the questionnaire items.

### Sample size estimation

For a confirmatory factor analysis, the sample size would be at least 10 subjects per item of the scale(s) to be tested [21]. The DRS-C-caregiver consisted of the highest number of items (i.e., 10 items), and thus about 120 family dyads were required after taking account of a potential non-response and/or incompletion rates of 17% [22]. This sample size could allow the achievement of a study power of 0.80 at 5% significance level, with a moderate correlation between dyadic relationship and self-efficacy in caregiving [23].

### Data collection

After obtained the research ethics approval from the Survey and Behavioural Research Ethics Committee of the Chinese University of Hong Kong (Reference No. SBRE-18-677), the researcher confirmed the eligibility of the participants during home visits. Informed written consent was obtained from all individual participants, with assurance of confidentiality and anonymity of data and right to withdrawal, before collecting any data. All data collected were anonymous, kept confidential, and used for research purposes only. Personal information or identities of the participants were not in any way identifiable in the papers. The research data were stored safely in a locked cabinet. The personal data were kept for six years after the study, after which the researcher would destroy it.

### Dyadic Relationship Scales (Chinese version, DRS-C)

The quality of dyadic relationship between family dyads was measured by the translated Chinese version of the DRS-C scales (DRS-C-patient and DRS-C-caregiver), which was self-rated on a four-point Likert-type scale ranging from 0 (strongly agree) to 3 (strongly disagree). A higher scale score indicated a worse dyad relationships [15] (Additional file 1).

### Hypertension self-care profile (HBP SCP)

The Chinese version of the HBP SCP-self-efficacy scale was used to measure hypertensive patients’ self-efficacy in hypertension management. The self-efficacy scale consisted of 20 items; each rated on a four-point Likert scale ranging from 4 (very confident) to 1 (not confident). A higher score indicates a higher level of perceived self-efficacy in hypertension management [24]. The Chinese version of HBP SCP revealed good psychometric properties in Chinese with hypertension with a Cronbach’s alpha coefficient of 0.93, and a moderate correlation between the HBP SCP and Treatment Adherence Questionnaire for Hypertension (TAQPH), r = 0.45 and 0.65 respectively, all *p* < 0.001 [25].

### Zarit burden interview (ZBI)

ZBI was used to measure the family carers’ caregiving burden [26]. The 22-item ZBI was a self-reporting instrument with a five-point Likert-type scale ranging from 0 (nerve) to 4 (nearly always); and a higher score indicated a greater perceived burden. It was valid and useful in Chinese dementia population [27], with a good intraclass correlation coefficient of 0.89 and split-half correlation coefficient of 0.87, as well as significant correlations with the Geriatric Depression Scale (r = 0.57, *p* < 0.001) and Hamilton Anxiety Scale (r = 0.44, *p* = 0.003).

### Measurement of blood pressure (BP)

A well-validated electronic upper-arm sphygmomanometer (OMRON HEM-752), the results of the validation have been published [28], was used to measure one’s BP. The method or procedure for measuring BP followed the international and national hypertension management guidelines [1, 2]. BP measurements (on upper arm) were repeated at an interval of 1 to 2 min; and the mean value of the two readings was recorded. If the difference between these two readings of SBP/DBP were more than 5 mmHg, the measurements would be repeated once; and an average value of the three readings would be recorded [2].

### Statistical analysis

The IBM SPSS version 25.0 and AMOS (IBM Crop. Armonk, NY) was employed for data analyses. Frequencies and percentages were calculated for categorical variables; and mean and standard deviations were for continuous variables. The missing values, which could not be replenished by reviewing the raw data, were replaced by the means imputation strategy. Since parametric tests (e.g., Pearson’s product-moment correlation and independent-sample t-test) were calculated to determine convergent and discriminant validity of the two DRS-C scales. The total scores of the scales should meet the statistical assumptions of normality, linearity and homoscedasticity [17]. Q-Q plot, skewness and kurtosis statistics were performed to examine the normality of the scores of DRS-C-patient, DRS-C-caregiver and self-efficacy subscale of the HBP SCP and ZBI. To those that were originally skewed, logarithm transformation was performed [29]. In this study, the scores of the DRS-C-patient and self-efficacy of HBP SCP were originally normally distributed, whereas the normality of the DRS-C-caregiver and ZBI scores was met with logarithm transformation. All statistical analyses were two-sided, and any *p* value of < 0.05 was considered statistically significant.

### Validity

#### Confirmatory factor analysis (CFA)

This study employed the CFA to confirm whether the constructs of DRS-C were similar to the original English versions in American communities in which two-factor solutions (positive dyadic interaction and dyadic strain) of the two DRS scales were found in both exploratory (EFA) and confirmatory factor analysis (CFA) [15]. Therefore, the two-factor solution in the two DRS-C scales was tested to see whether they were similar to the original DRS versions. The Maximum Likelihood Estimation for the CFA was performed to test the model fit. The model-fit indices and criteria [30, 31], including root-mean-square error of approximation (RMSEA), comparative fit index (CFI), goodness-of-fit index (GFI), Tucker and Lewis Index (TLI), and standardized root-mean-square residual (SRMR), were adopted. RMSEA values at 0.05 or lower would indicate a good model fit, and those ranging from 0.05 to 0.08 would represent a moderate fit. The GFI, CFI and TLI values at 0.95 or above could indicate a well-fitting model, and those between 0.90 and 0.95 would indicate an acceptable fit. In addition, a SRMR value at 0.08 or lower could be indicative of a good model-fit.

#### Convergent validity

Convergent validity was examined by hypothesis-testing approach. The efficacy scale in Hypertension Self-Care Profile (HBP SCP) was employed in the evaluation of convergent validity of the DRS-C-patient. The Shared Care Model posited that family dyadic relationship in daily care could influence patients’ self-care efficacy [32]. Previous studies have also demonstrated the improved dyadic relationship might lead to positive effects on self-efficacy of patients with different heart diseases [33–35]. As such, a positive correlation between the mean scores of the DRS-C-patient and self-efficacy scale of the HBP SCP would be tested to provide evidence on the convergent validity of the DRS-C-patient.

The convergent validity of the DRS-C-caregiver was assessed by correlation test between the mean scores of the scale (DRS-C-caregiver) and Zarit Burden Interview (ZBI) scale. The ZBI has been widely used to assess the subjective burden of caregivers. The Pearlin Stress Process Model proposes that stress factors, including dyad strain, caregiver’s role captivity, role strain, and perceived stress, can determine caregiver burden [36]. Therefore, a correlation between the mean scores of the DRS-C-caregiver and the ZBI with similar construct or conceptualisation can provide evidence for the convergent validity of the DRS-C-caregiver.

#### Known-groups validity

The known-groups validity of the DRS-C was examined using known-groups comparison test. Based on the available empirical evidence [12], patients with a better dyadic relationship would exhibit better levels of self-care and self-efficacy of lifestyle behaviours and medication adherence, which could determine the people with hypertension’ BP values [2]. Therefore, the patients with well-controlled BP in normal level (SBP < 140 mmHg and DBP < 90 mmHg) were hypothesized to have a significantly better dyadic relationship than those without controlled normal BP as tested by independent-sample t-test.

### Reliability

#### Internal consistency

The internal consistencies of DRS-C scales were examined using Cronbach’s alpha statistics and item correlation analysis. Cronbach’s alpha of 0.7 or higher indicates an acceptable reliability [37]. Item correlation analysis of DRS-C scales was performed using corrected item-to-total correlation and Cronbach’s alpha statistics with item deletion(s). A corrected item-to-total correlation coefficient of at least 0.3 is the acceptable level of homogeneity of individual items to the scale/subscale [38]. The inconsistent item could be considered to be deleted if its corrected item-total correlation was less than 0.3; and its deletion would not cause a decrease of 0.1 or more in the Cronbach’s α coefficient of the scale [39].

### Stability

The weighted Kappa statistic was computed to examine the test–retest reliability of the DRS-C scales at a two-week retest interval [40]; and the intraclass correlation coefficient (ICC) ≥ 0.7 can indicate a satisfactory stability [41].

## Results

### Phase one: results on translation, face and content validity and semantic equivalence

In the translation and back-translation of the DRS scales, several inconsistencies in meanings of a few terminologies or words were found in the back-translated versions when compared to the original English versions, including (1) The “*strongly* agree” and “*strongly* disagree” in the original English version translated into “*totally* agree” and “*totally* disagree”; (2) the word “*about*” in “I have learned some good things *about* my relative”, “I have learned some good things about myself”, and “I have learned some nice things *about* other people in my life” turned into “*from*”; (3) “*resentful*” in “I felt *resentful* toward her/him” turned into “*unsatisfied*”. These items in question were again translated and blindly back-translated by the third and fourth translators, respectively. There were no discrepancies or differences in meanings between the other items of the two DRS scales found. The Chinese version of DRS (DRS-C) was then finalised for equivalence testing.

In testing face validity of the two translated scales, no item was rated less than 3 by > 4 panel members; and hence, there was no amendment needed for all the items of the scales.

For content validity testing, all items were rated as 3 or 4 by four or all (> 75%) of the five expert panel members, except the item of ‘*I have learned some nice things about other people in my life*’ rated by three members (60%) as 2 (I-CVI = 0.4). This item in both scales was finally deleted based on its inconsistency with other items in terms of low Cronbach’s alpha and item-scale correlation in later reliability and validity testing in Phase Two. After deleting the item, the Chinese versions of the DRS-C-patient and DRS-C-caregiver consisted of 9 and 10 items, respectively. The I-CVI ranged from 0.8 to 1.0 for both translated versions and the S-CVIs were both at 0.98, indicating a very satisfactory content validity of the two scales.

### Phase two: results of testing the psychometric properties of the DRS-C scales

#### Characteristics of participants

There were 132 family dyads (patients and their family carers) with hypertension recruited. All participants completed the data collection. Their average SBP was 149.35 ± 19.01 mmHg and average DBP was 82.11 ± 13.33 mmHg. About one-third (around 29.55%) of the patients (n = 39) had controlled ‘normal range’ BP (e.g., SBP < 140 mmHg and DBP < 90 mmHg). More than half (n = 80, 60.61%) of the family carers recruited were the patients’ spouse. More details of the sociodemographic characteristics of the participants are summarized in Table 1.

**Table 1:** Socio-demographic and clinical characteristics of people with hypertension (*N* = 132)

Characteristics	Mean ± SD or *n* (%)
People with hypertension	
Age (Range: 30 to 94)	66.80 ± 12.22
Gender	
Male	52 (39.39)
Female	80 (60.61)
Marital status	
Married	98 (74.24)
Single/separated/divorced/widowed	34 (25.76)
Employment nature	
Farmer	129 (97.73)
Others (e.g., businessman and public official)	3 (2.27)
Educational level	
Illiteracy	31 (23.48)
Primary school	88 (66.67)
Secondary or above	13 (9.85)
Duration of hypertension (range: 1 to 30 years)	8.47 ± 5.58
SBP (mmHg)	149.35 ± 19.01
DBP (mmHg)	82.11 ± 13.33
Patients with controlled normal BP^*^	39 (29.55)
Number of comorbidities	1.64 ± 0.632
Comorbidities	
Diabetes	43 (32.58)
Arthrophlogosis	19 (14.39)
Coronary heart disease	15 (11.36)
Others^$^	27 (20.45)
Antihypertensive drugs intake	
Yes	106 (80.30)
No	26 (19.70)
Relationship of family carer with patient	
Spouse	80 (60.61)
Son/son in-law/daughter/daughter in-law	49 (37.12)
Other family members	3 (2.27)
*Family carers*	
Age (range: 28 to 83)	57.68 ± 11.49
Gender	
Male	82 (62.12)
Female	50 (37.88)
Employment nature	
Farmer	127 (96.21)
Others (e.g., teacher and businessman)	5 (3.79)
Educational level	
Illiteracy	12 (9.09)
Primary school	94 (71.21)
Secondary or above	26 (19.70)

^#^Family carers helped patients in medication taking, blood pressure monitoring, clinic visits, smoking cessation, alcohol control, weight loss, healthy diet, sodium restriction, and/or physical activity

^$^Other comorbidities were kidney stones, alcoholic hepatitis, and chronic bronchitis, etc.

^*^SBP < 140 mmHg and DBP < 90 mmHg

### Confirmatory factor analysis (CFA)

**Fig. 1:**
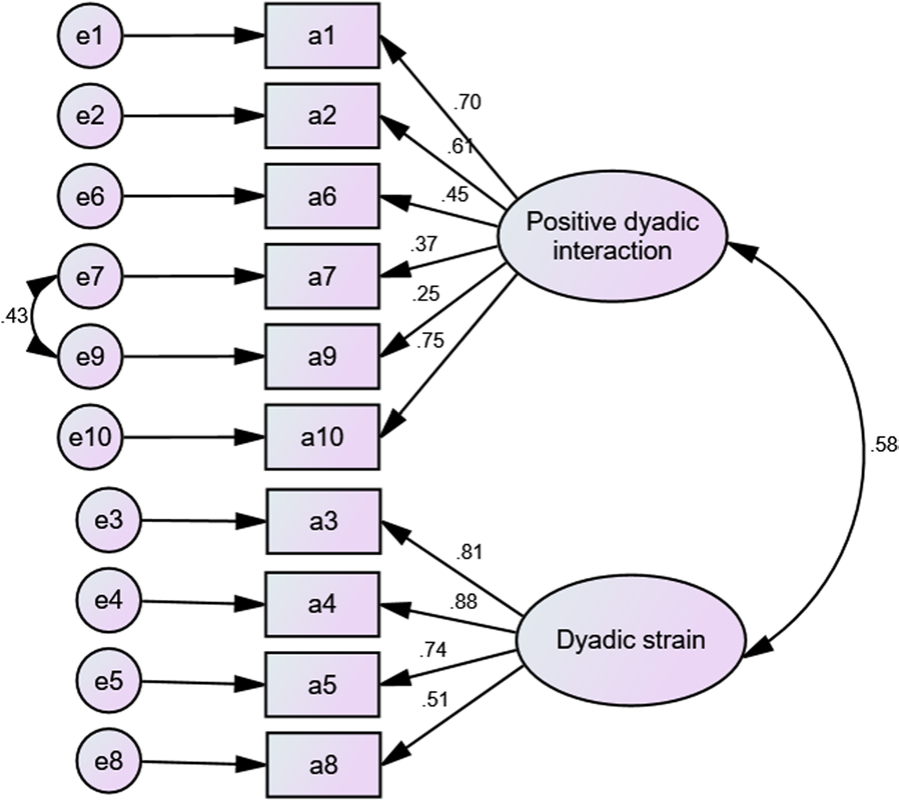
Confirmatory factor analysis of Dyadic Relationship Scale—patient version (DRS-C-patient)

**Fig.2:**
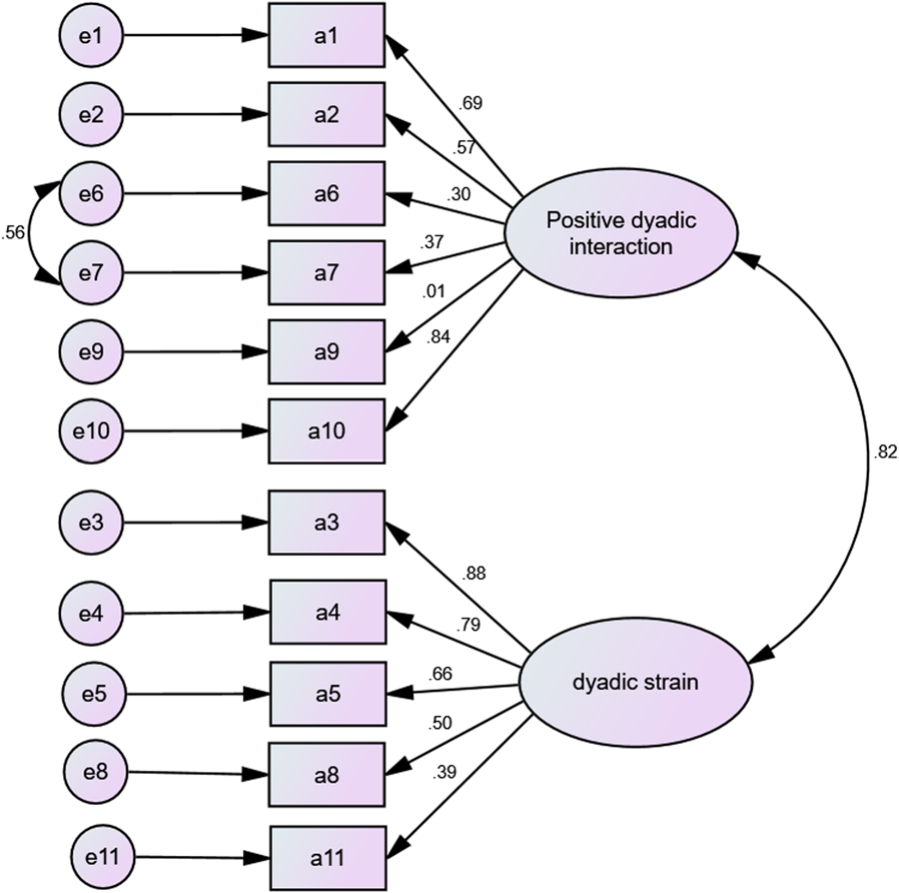
Confirmatory factor analysis of Dyadic Relationship Scale—caregiver version (DRS-C-caregiver)

To improve the fit indices, according to modification indices, a post hoc model modification was performed. Then the model was adjusted by constructing paths between the residuals (e.g., e7 and e9 in Fig. 1, e6 and e7 in Fig. 2). The CFA was computed and a well-fitting model of DRS-C-patient was identified with χ2/df = 1.47, *p* = 0.04, RMSEA = 0.06, GFI = 0.941, CFI = 0.961, TLI = 0.947, SRMR = 0.019 (Fig. 1). A well-fitting model of DRS-C-caregiver was identified with χ2/df = 1.340, *p* = 0.092, RMSEA = 0.039, GFI = 0.940, CFI = 0.975, TLI = 0.965, SRMR = 0.014 (Fig. 2).

#### Internal consistency and stability of DRS-C-patient

The corrected item-to-total correlation coefficients of individual items in DRS-C-patient ranged from 0.40 to 0.65, which met the suggested criteria level (≥ 0.3) except item 9 (I have learned some nice things about other people in my life) with the correlation of 0.28 (Table 2). Therefore, item 9 was considered as non-homogeneous with other items in DRS-C-patient and could be deleted. The deletion of this item caused an increase of 0.005 for the Cronbach’s alpha value for the overall scale, from 0.816 to 0.821, and thus, item was deleted from DRS-C-patient. With one item deleted, the Cronbach’s alpha value of the nine-item DRS-C-patient was 0.82, which supported the internal consistency of the DRS-C-patient.

**Table 2:** The item analysis and weighted kappa statistics of the DRS-C-patient and DRS-C-caregiver (N = 132)

Item no	DRS-C-patient		DRS-C-caregiver	
	Corrected item-to-total correlation	Cronbach’s alpha with item deletion	Corrected item-to-total correlation	Cronbach’s alpha with item deletion
1	0.52	0.80	0.57	0.79
2	0.45	0.80	0.53	0.80
3	0.62	0.79	0.70	0.77
4	0.65	0.78	0.61	0.79
5	0.58	0.79	0.61	0.79
6	0.40	0.81	0.35	0.81
7	0.40	0.81	0.32	0.82
8	0.51	0.80	0.46	0.80
9	0.28	0.82	0.68	0.78
10	0.55	0.80	0.05	0.83
11	–	–	0.31	0.81

The two-week test–retest reliability of the nine-item DRS-C-patient, with ICC = 0.97 (*p* < 0.001) for the overall scale, indicating very satisfactory stability.

#### Internal consistency and stability of DRS-C-caregiver

The corrected item-to-total correlation coefficients of items in the DRS-C-caregiver were 0.31 to 0.70, which were all ≥ 0.3 except item 10 with the correlation of 0.05 (see Table 2). Therefore, item 10 was considered as a non-homogeneous item in the DRS-C-caregiver. The deletion of this item caused an increase of 0.016 on the Cronbach’s alpha value for the overall scale, from 0.82 to 0.83, and thus, item was deleted. With this single item deleted, the Cronbach’s alpha value of the 10-item DRS-C-patient was 0.83, which supported the internal consistency of the DRS-C-caregiver was satisfactory.

The two-week test–retest reliability of the 10-item DRS-C-caregiver was very satisfactory, with ICC = 0.96 (*p* < 0.001) for all the items.

The CFA for DRS-C-caregiver and DRS-C-patient was repeated after item delection. The DRS-C-patient with item delection was also well-fitted to the original model, with χ2/df = 1.28, *p* = 0.16, RMSEA = 0.047, GFI = 0.951, CFI = 0.981, TLI = 0.972, SRMR = 0.015; and the items loading ranged from 0.37 to 0.88. After deleteing one item, the DRS-C-caregiver was also well-fitted, χ^2^/df = 1.49, *p* = 0.043, RMSEA = 0.059, GFI = 0.935, CFI = 0.965, TLI = 0.953, SRMR = 0.014. The items loading ranged from 0.30 to 0.88.

### Convergent validity

A significant negative correlation between the mean scores of the DRS-C-patient and HBP SCP (Pearson’s r = − 0.70, *p* < 0.001) provided support for a very satisfactory convergent validity of the DRS-C-patient. A satisfactory convergent validity of the DRS-C-caregiver was also identified by a significant positive correlation between the mean scores of the DRS-C-caregiver and ZBI (Pearson’s r = 0.79, *p* < 0.001).

### Known-groups validity

The results of the known-groups comparison supported good know-groups validities of both the DRS-C-patient and DRS-C-caregiver. The mean scores of the DRS-C-patient and DRS-C-caregiver of the patients with controlled normal BP were 6.38 (SD = 1.16) and 8.41 (SD = 1.65), respectively; whereas, the mean scores of those patients without controlled normal BP control were 8.47 (SD = 1.40) and 12.87 (SD = 2.97), respectively. Significant differences were found between the group of people with hypertension with controlled normal BP and the group without controlled normal BP on the DRS-C-patient scores (t = − 8.10, *p* < 0.001), and the DRS-C-caregiver scores (t = − 9.15, *p* < 0.001).

## Discussion

Our study showed that the Chinese version of DRS was a reliable and valid instrument for assessing the dyadic relationship quality in people with hypertension in the rural China communities.

A cross-cultural validation as such in this study should be performed before the use of an instrument in a new language/culture. The Chinese are usually more family oriented and interdependent and strong in filial responsibilities; whereas, the westerners (e.g., Americans) are more individual-oriented and independent [20]. Influenced by Confucianism, ‘filial piety’ is the core of Chinese family values. Caring for a sick family member is not only a family duty but also a moral imperative [14]. In this study, the results of semantic equivalence computed by a group of bilingual participants and face validation performed by 10 family dyads supported the basic level of cultural relevance of the DRS-C scales to the Chinese rural population/culture.

The findings also support the high internal consistencies (Cronbach’s alpha = 0.82 and 0.83, respectively) and test–retest reliability (ICC = 0.97 and 0.96, respectively) of DRS-C-patient and DRS-C-caregiver. Therefore, the DRS-C scales demonstrated very satisfactory reliability in the assessment of dyadic relationship quality among family dyads, similar to the original DRS scales [15].

The confirmatory factor analysis (CFA) verified the original two-factor structure (including positive dyadic interaction and dyadic strain) of the DRS-C-patient and DRS-C-caregiver in American population [15]. The concept of family care intrinsically involves patients and their family carers (i.e., the dyad) in close relationships, which could lead to positive or negative interpersonal interactions between family members. Recent systematic reviews have demonstrated that positive dyadic interactions in family care can exert positive effects on patients’ and family’s health outcomes [3, 4]. Nevertheless, recent research indicated that family members involving in chronic illness care can produce very negative interactions which could negatively affect the relationships between people with hypertension and their family members, and thus contribute to deteriorations of their physical and psychological health [10, 12]. The level or change of quality of the family dyadic relationship measured with the DRS-C can be a good indicator of the success in family caregiving, and thus hypertension care by family caregivers to their patients.

The DRS-C-patient and DRS-C-caregiver had satisfactory convergent and known-groups validities in Chinese hypertensive family dyads. The positive correlations between the DRS-C-patient and self-efficacy in hypertension care (HBP SCP) scores, and between the DRS-C-caregiver and family burden (ZBI) scores, supported convergent validities of the two scales. The two significant correlations indicated that the two DRS-C scales were valid in measuring family dyadic relationship (from the perspectives of both patients and caregivers) in caregiving process of people with hypertension. The convergent validity result of the DRS-C-patient was consistent with the theoretical assumption and with research evidence on association between family dyadic relationship and self-efficacy in people with heart diseases [33–35]. Meanwhile, the significant correlation between the family caregivers’ perceived dyadic relationship and burden of care were consistent with this the assumption that family carers had better family dyadic relationship (i.e., higher positive interaction and lower dyadic strain) exhibited less subjective perceived burden in caregiving [36].

Satisfactory known-groups validities of the DRS-C scales were obtained from the findings of significant differences in DRS-C-patient and DRS-C-caregiver scores between the group of people with hypertension with controlled normal BP and the group without controlled normal BP. The findings were consisted with the available empirical evidence, in which patients with a better dyadic relationship would show better levels of lifestyle modifications and medication adherence, resulting in better BP values [12].

### Limitations

There are several limitations of this study. Firstly, the participants were recruited in one rural village of one (Hunan) of 34 provinces of China. Convenience sampling at one site further reduce the generalizability of the findings. Secondly, only people with hypertension were included in the study, the psychometric properties of the DRS-C scales in other chronic illnesses (e.g., stroke and heart diseases) populations are uncertain. Thirdly, the sample size of this study was relatively small. Small sample size could affect the quality of model fit indices. For example, the factor loadings of items 7 and 10 in the DRS-C-caregiver were lower than those obtained in the American study with a sample size of 200.

### Recommendations for future research

Another larger-scale validation study should be conducted with a diverse population of hypertension and/or other chronic illnesses in different geographical areas of China. Other reliability and validity tests can be included in future research, such as inter rater reliability, divergent or discrimination validity, sensitivity and specificity to hypertension care, to enrich the understanding of the psychometric properties of the scales before use.

## Conclusion

Overall, the DRS-C-patient and DRS-C-caregiver can be research instruments with sound psychometric properties to measure dyadic relationship quality of Chinese people with hypertension and their family carers in the caregiving process, particularly in the rural communities. The Chinese versions would be used to evaluate the effectiveness of a family support programmes for people with hypertension living in China on their quality of dyadic relationship in research and clinical practice.

## Supplementary Information

**Additional file 1:** The Dyadic Relationship Scale.

## Data Availability

The datasets used and/or analysed of this study available from the corresponding author on reasonable request.

## Abbreviations

DRS: Dyadic Relationship Scale
S-CVI: Scale content validity index
I-CVI: Item content validity index
HBP SCP: Hypertension self-care profile
ZBI: Zarit burden interview
CFA: Confirmatory factor analysis
RMSE: Root-mean-square error of approximation
CFI: Comparative fit index
GFI: Goodness-of-fit index
TLI: Tucker and Lewis index
SRMR: Standardized root-mean-square residual
