# Supplementary material for: Testing the psychometric properties of a Chinese version of Dyadic Relationship Scale for families of people with hypertension in China
Source: BMC Psychol. 2022 Feb 21;10:34. doi: 10.1186/s40359-022-00747-2 (PMC8862279; doi:10.1186/s40359-022-00747-2)
Supplement: Supplementary file 1 — Additional file 1: The Dyadic Relationship Scale. [file 40359_2022_747_MOESM1_ESM.pdf]

Dyadic Relationship Scale (Caregiver)

This series of questions address some of the difficulties that people face as they care for a relative. I'd like to talk about how helping your [REL] has affected your relationship with her/him during the past month. [HAND CG CARD]. Please refer to this card for your responses. How much do agree or disagree with the following statements?

**Because of helping my [REL]:**	**Strongly Agree**	**Agree**	**Disagree**	**Strongly Disagree**
a. I felt closer to her/him than I have in awhile	0	1	2	3
b. I have learned some good things about my [REL]	0	1	2	3
c. I felt angry toward her/him	3	2	1	0
d. I felt depressed because of my relationship with her/him	3	2	1	0
e. I felt resentful toward her/him	3	2	1	0
f. I have had more patience than I have had in the past	0	1	2	3
g. I have learned some good things about myself	0	1	2	3
h. I felt that my relationship with her/him was strained	3	2	1	0
i. I have learned some nice things about other people in my life	0	1	2	3
j. Communication between my [REL] and me has improved	0	1	2	3
k. I felt that s/hhe madee requests over and above what s/he needed	3	2	1	0

Dyadic Relationship Scale (Care Recipient)

I'd like to talk about how your memory problems have affected your relationship with your [CG] over the <u>past month</u>? **[HAND RESP CARD]**. Please refer to this card for your responses and again, we are talking about the <u>past month</u>. How much do you agree or disagree with the following statements?

[For Care Recipients who are unabe to distinguish between the four response categories, use only the **"Yes"** and **"No"** response options and the reverse of RESP CARD which has the appropriate "Yes" "No" responses for the **<u>entire</u>** scale]

**Because of helping my [REL]:**	**Strongly Agree**	**Agree**	**Disagree**	**Strongly Disagree**
a. I felt closer to her/him than I have in awhile	0	1	2	3
b. I have learned some good things about myself	0	1	2	3
c. I felt angry toward [CG]	3	2	1	0
d. I felt depressed because of my relationship	3	2	1	0
e. I felt resentful toward my [CG]	3	2	1	0
f. I have had more patience than I have had in the past	0	1	2	3
g. I have learned some good things about my [CG]	0	1	2	3
h. I felt that my relationship with my [CG] was strained	3	2	1	0
i. I have learned some nice things about other people in my life	0	1	2	3
j. Communication between my [REL] and me has improved	0	1	2	3
